# A predominance of hypertensive heart disease among patients with cardiac disease in Buea, a semi-urban setting, South West Region of Cameroon

**DOI:** 10.1186/s13104-017-3034-6

**Published:** 2017-12-04

**Authors:** Clovis Nkoke, Christelle Makoge, Anastase Dzudie, Liliane Kuate Mfeukeu, Engelbert Bain Luchuo, Alain Menanga, Samuel Kingue

**Affiliations:** 1 Buea Regional Hospital, Buea, South West Region Cameroon; 2 Faculty of Medicine and Biomedical Sciences, University of Yaounde 1, Yaounde, Cameroon; 3 Douala General Hospital, Douala, Cameroon; 4 Athena Institute for Research on Innovation and Communication in Health and Life Sciences, Vrije Universiteit, Amsterdam, The Netherlands

**Keywords:** Cardiac disease, Hypertensive heart disease, Rheumatic heart disease, Cameroon

## Abstract

**Objective:**

The pattern of heart disease is diverse within and among world regions. The little data on the spectrum of heart disease in Cameroon has been so far limited to major cities. We sought to describe the pattern of heart disease in Buea, the South West Region of Cameroon, a semi-urban setting. This was a descriptive cross-sectional study. Between June 2016 and April 2017 the echocardiography register of the Buea Regional Hospital was surveyed. We extracted data on the age, sex and echocardiographic diagnosis.

**Results:**

Out of 529 patients who underwent echocardiography, 239 (45.2%) had a definite heart disease. There were 137 (57.3%) females. The mean age was 58 years (range 3–94 years). The most common echocardiographic diagnoses were hypertensive heart disease (43.2%), dilated cardiomyopathies (17.6%), ischemic heart diseases (9.6%), and cor pulmonale (8.8%). Rheumatic heart disease affected 6.7% of the patients. The most common rheumatic heart disease was mitral stenosis followed by mitral regurgitation. Congenital heart disease represented 2.1% and 5 patients (2.1%) had pulmonary hypertension. Hypertensive heart disease is the most common cardiac disease in this semi-urban region in Cameroon. Rheumatic heart disease still affects a sizable proportion of patients. Prevention of cardiac disease in our setting should focus on mass screening, the treatment and control of hypertension.

## Introduction

Sub-Saharan Africa like other developing countries of the world is undergoing epidemiological transition with increase in the prevalence of non-communicable diseases including cardiovascular diseases [1, 2]. Cardiovascular diseases are the leading cause of death worldwide with more than 80% of the deaths occurring in low and middle income countries including sub-Saharan Africa [3].

The pattern of heart disease is diverse within and among world regions. The pattern of heart disease can provide an indicator of the health transition from communicable to non-communicable diseases. There are few studies describing the pattern of heart disease in Cameroon where cardiovascular specialists and equipements for the diagnosis of heart diseases are very limited and located mainly in the big urban cities. Whether data generated through these studies reflect rural and semi-urban area is unknown. The aim of this study was to report the pattern of heart disease in the South West Region of Cameroon, a semi-urban setting.

## Main text

### Methods

#### Study design and setting

This was a descriptive cross-sectional study. The study was performed at the cardiac exploration unit of Buea Regional Hospital. It is the referral hospital in the South West Region of Cameroon. The town has a population of about 130,000 inhabitants. This region is characterized by a very limited access to effective interventions for prevention, diagnosis and treatment of cardiovascular diseases. In 2016, a cardiologist was posted to this hospital. The hospital receives patients referred from other parts of the region for the investigation and/or management of suspected heart disease. Before that, patients with suspected heart disease had to travel to other regional headquarters to see a cardiologist.

The echocardiography register was surveyed between June 2016 and April 2017. Only the first echocardiographic examination report for each patient was included. Using a pre-defined questionnaire, we extracted data on age at the time of diagnosis, sex, and the echocardiographic diagnosis.

Echocardiographic examination was performed in the parasternal long axis, short axis, apical four chamber and occasionally in the subcostal and suprasternal views. Indices analyzed included the left ventricle telesystolic diameter (LVIDS), left ventricle telediastolic diameter (LVIDD) and the ejection fraction (EF). All the echocardiographic diagnoses were based on existing guidelines.

The diagnosis of rheumatic heart disease (RHD) was based on the World Heart Federation (WHF) criteria for echocardiographic diagnosis of RHD. Briefly, RHD was defined by the presence of any evidence of mitral or aortic regurgitation seen in two planes associated with at least two of the following morphologic abnormalities of the regurgitating valve: restricted leaflet motility, focal or generalized valvular thickening, and abnormal sub-valvular thickening [4].

Hypertensive heart disease was diagnosed in the presence of any or combination of the following abnormalities: left ventricular diastolic dysfunction (e.g. altered E:A ratio), left ventricular hypertrophy (indexed LV mass > 51 g/m^2.7^), left ventricular systolic dysfunction and dilated left atrium, a surrogate of impaired LV filling (left atrial diameter > 3.8 cm in women and > 4.2 cm in men) in the presence of hypertension. Left ventricular geometric patterns were defined according to Ganau et al. [5].

Ischemic heart diseases were documented by detection of regional wall motion abnormality on different region of heart (such as loss systolic thickening, hypokinesia, akinesia dyskinesia) and associated with LV systolic dysfunction.

Dilated cardiomyopathy was diagnosed when there are dilated heart chambers with normal or decreased wall chambers as well as impaired LV systolic function [6].

Pericardial effusion was diagnosed when there is echo free space between the visceral and parietal pericardium.

Cor pulmonale was present when there is dilated and hypertrophied right ventricle (RV), evidence of increased RV systolic pressure D-shaped LV in diastole (diastolic flattening of the LV septum).

#### Data analysis

The data collected was analyzed using SPSS software version 20. Continuous variables were expressed as mean ± SD (standard deviation) and categorical variables expressed as percentages. Differences in categorical variables were assessed by Chi square analysis where appropriate. A p value of < 0.05 was considered statistically significant.

### Results

**Fig. 1:**
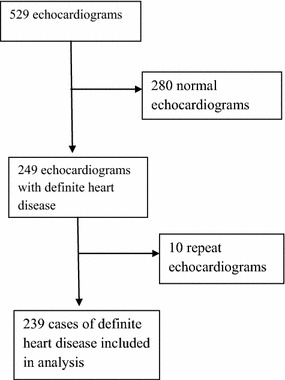
Flow diagram for cases selection

**Fig. 2:**
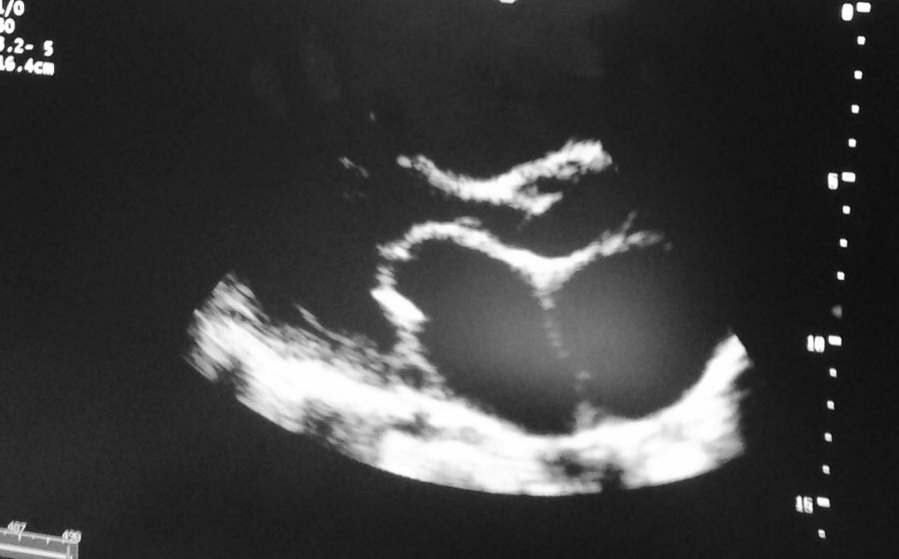
Echocardiogram of a subject with rheumatic mitral stenosis

**Fig. 3:**
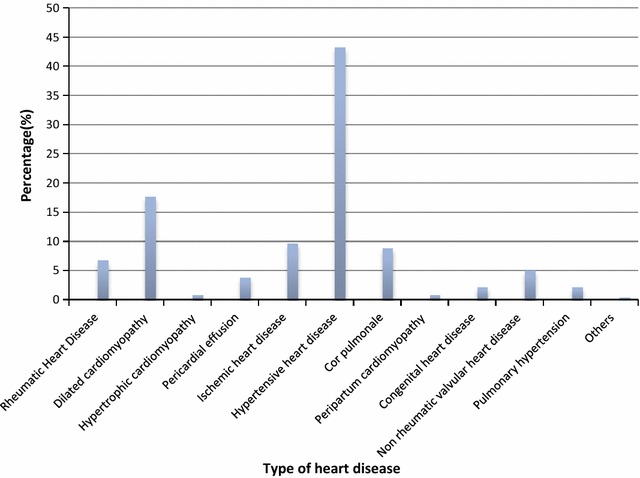
Distribution of different heart diseases

**Fig. 4:**
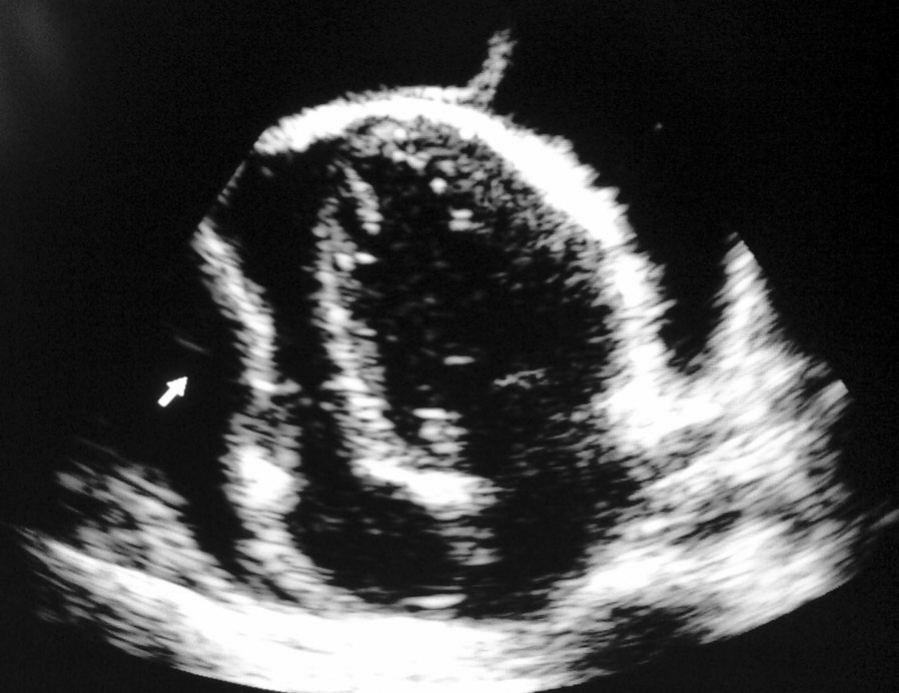
Echocardiogram of a subject with pericardial effusion

During the 11 month period, 529 echocardiograms were performed (Fig. 1). There were 239 echocardiograms with a definite heart disease. There were 137 (57.3%) women. The mean age of all the patients was 58.0 years ± 15.8 SD and ranged from 3 to 94 years. Women were significantly older than men (58.8 vs 56.9 years; p = 0.011). The most common conditions were hypertensive heart disease (43.2%), dilated cardiomyopathy (17.6%), and ischemic heart disease (9.6%), Table 1. Rheumatic heart disease accounted for 6.7% of heart diseases. Figure 2 shows a subject with rheumatic mitral stenosis. There were 5 (2.1%) cases of congenital heart disease. Congenital heart diseases included tetralogy of fallot (20%), atrial septal defect (20%) and ventricular septal defect (60%). Among the patients with dilated cardiomyopathy, 6 were HIV positive. Table 2 shows the different types of valvular lesions. Figure 3 shows the distribution of different heart diseases and Fig. 4 shows a subject with pericardial effusion.

**Table 1:** Echocardiographic diagnosis in the 239 subjects by gender

Heart disease	Male, (n)	Female, (n)	Total (%)
Rheumatic heart disease	4	12	16 (6.7)
Dilated cardiomyopathy	22	20	42 (17.6)
Hypertrophic cardiomyopathy	2	0	2 (0.8)
Pericardial effusion	4	5	9 (3.8)
Ischemic heart disease	12	11	23 (9.6)
Hypertensive heart disease	37	64	101 (43.2)
Cor pulmonale	7	14	21 (8.8)
Peripartum cardiomyopathy	0	2	2 (0.8)
Congenital heart disease	3	2	5 (2.1)
Non rheumatic valvular heart diseases	8	4	12 (5.0)
Pulmonary hypertension	2	3	5 (2.1)
Others	1	0	1 (0.4)
Total	102	137	239

**Table 2:** Valvular lesions

Valvular lesion	Frequency, (n)	Percentage (%)
Rheumatic MS	7	25
Rheumatic MR	5	18
Rheumatic MR + MS	2	7.1
Rheumatic AR	1	3.6
Rheumatic AS	1	3.6
Degenerative AS	2	7.1
Mitral valve prolapse	2	7.1
Degenerative AR	7	25

*MS* mitral stenosis, *MR* mitral regurgitation, *AR* aortic regurgitation, *AS* aortic stenosis

### Discussion

We have reported on the spectrum of cardiac disease for the first time in the South West Region of Cameroon, a semi-urban setting. This echocardiographic hospital based study has shown that hypertensive heart disease is by far the most common type of heart disease followed by dilated cardiomyopathy and ischemic heart disease.

Hypertensive heart disease was the most common heart disease in this study which could be expected given the high prevalence of hypertension in the general population coupled with poor awareness, low treatment control and control rates [7, 8]. In a study conducted by Jingi et al. looking into the pattern of heart disease in the West Region of Cameroon, hypertensive heart disease was the most prevalent condition accounting for 41.5% of cardiac diseases diagnosed by echocardiography [9]. This report is comparable to the finding in our study. In similar studies in Nigeria, it was reported that hypertensive heart disease was the most common form of heart disease diagnosed on echocardiography [10–12]. We could not tell if there was an association between the duration and severity of hypertension and the development of hypertensive heart disease.

It is well known that hypertension forms the bulk and is the foundation of cardiovascular diseases in Africa. The Abuja Heart Study (2006–2010) in Nigeria [13] and the Heart of Soweto Study (2006–2008) in South Africa [14] showed that hypertension is now a dominant cause of heart failure in adults in these countries. In the THESUS-HF registry, hypertension accounted for 43.9% of heart failure in sub-Saharan Africa [15]. In a major urban city of Cameroon (Yaounde), Kingue et al. reported that hypertension accounted for 54.49% of causes of heart failure [16]. In Cameroon, hypertension has become a major public health problem. It is estimated to affect about 30% of the general population [7]. This is as a result of the epidemiological transition Cameroon is traversing like other developing countries. In a survey of blood pressure control among patients with hypertension in Yaounde, the capital city of Cameroon, only 30% of patients had their blood pressure controlled [8].

Globally, hypertension is the leading cause of cardiovascular diseases and deaths, and accounts for about 7.5 million deaths per year [17]. Like most cardiovascular diseases, the natural course of hypertension can be modified with the use of effective and inexpensive medications. Many randomized controlled trials have demonstrated unequivocally that treatment of hypertension reduces the risk of stroke, coronary heart disease, congestive heart failure and mortality [18, 19]. It is therefore imperative that lowering of blood pressure to targets be achieved in patients with hypertension to prevent heart disease and other cardiovascular diseases.

Dilated cardiomyopathy was the second most common heart disease in our study, representing 17.9%. As also reported by Jingi et al. cardiomyopathies were the second most frequent cause of heart disease in the West Region of Cameroon [9]. Our findings are also similar to that reported by Kingue et al. in Yaounde [16]. Similar studies in sub-Saharan Africa have demonstrated cardiomyopathies to be a significant cause of heart disease [10, 15, 20, 21].

In our study, 9.8% of the patients had ischemic heart disease representing the 3rd most common heart disease. The proportion of ischemic heart disease in our study was higher than that reported by Jingi et al. (2.4%) in the West Region of Cameroon and Ogah et al. (0.6%) and Ukoh et al. (2.9%) in Nigeria [9, 10, 12]. Although ischemic heart disease was considered to be rare in sub-Saharan African, recent evidence suggests ischemic heart disease is by no means rare in Africans [22, 23]. This increasing incidence of ischemic heart disease in Africans is due to the epidemiological transition with the adoption of western lifestyles.

Rheumatic heart disease was a significant cause of heart disease in our study (6.8%). On the contrary, rheumatic heart disease accounted for only 3.4% of all cases of heart disease in the West Region of Cameroon [9]. Ukoh et al. in a similar study in Benin City in Nigeria had a prevalence of 18.1% for rheumatic heart disease [12]. Rheumatic disease has almost disappeared in developed countries but it remains a major public health issue in children and young adults in low and middle income countries including sub-Saharan Africa [24–27]. Rheumatic heart disease still remains a significant cause of heart failure in sub-Saharan Africa. In the THESUS-HF registry, it was the third most common cause of heart failure after hypertension and cardiomyopathies [15].

The spectrum of pericardial disease in our study was different from that reported by Jingi et al. in the West Region of Cameroon where they reported a higher proportion of pericardial disease [9].

### Conclusion

Hypertensive heart disease is the most common cardiac condition in this semi-urban setting. Effective preventive strategies of heart disease heart diseases in this setting should focus on detection, treatment and control of hypertension.

## Limitations

Our study is a hospital based review of prospectively recruited patients and subject to bias. Patients referred for echocardiography may be those with more severe patterns of heart disease as patients with more severe lesions are more likely to seek medical attention. Despite this shortcoming our study provides insight into the pattern of heart disease in this region of our country as seen on echocardiography.

## References

[1] Mathers CD, Loncar D (2006). Projections of global mortality and burden of disease from 2002 to 2030. PLoS Med..

[2] Caldwell JC (2001). Population health in transition. Bull World Health Organ.

[3] World Health Organization (2009). World health statistics 2009.

[4] Remenyi B, Wilson N, Steer A, Ferreira B, Kado J, Kumar K, Lawrenson J, Maguire G, Marijon E, Mirabel M (2012). World Heart Federation criteria for echocardiographic diagnosis of rheumatic heart disease—an evidence-based guideline. Nat Rev Cardiol..

[5] Ganau A, Devereux RB, Roman MJ, de Simone G, Pickering TG, Saba PS, Vargiu P, Simongini I, Laragh JH (1992). Patterns of left ventricular hypertrophy and geometric remodeling in essential hypertension. J Am Coll Cardiol..

[6] Cardiomyopathies: report of a WHO expert committee. World Health Organ Tech Rep Ser. 1984;697:7–64. 6428049

[7] Kingue S, Ngoe CN, Menanga AP, Jingi AM, Noubiap JJ, Fesuh B, Nouedoui C, Andze G, Muna WF (2015). Prevalence and risk factors of hypertension in urban areas of Cameroon: a nationwide population-based cross-sectional study. J Clin Hypertens (Greenwich)..

[8] Menanga A, Edie S, Nkoke C, Boombhi J, Musa AJ, Mfeukeu LK, Kingue S (2016). Factors associated with blood pressure control amongst adults with hypertension in Yaounde, Cameroon: a cross-sectional study. Cardiovasc Diagn Ther..

[9] Jingi AM, Noubiap JJ, Kamdem P, Wawo Yonta E, Temfack E, Kouam Kouam C, Kingue S (2013). The spectrum of cardiac disease in the West Region of Cameroon: a hospital-based cross-sectional study. Int Arch Med..

[10] Ogah OS, Adegbite GD, Akinyemi RO, Adenisa JO, Alabi AA, Udofia OI (2008). Spectrum of heart diseases in a new cardiac service in Nigeria: an echocardiographic study of 1441 subjects in Abeokuta. BMC Res Notes.

[11] Balogun MO, Urhoghide VA, Ukoh VA, Adebayo RA (1999). A preliminary audit of two-dimensional and Doppler echocardiographic service in a Nigerian tertiary private hospital. Nig J Med.

[12] Ulasi II, Arodiwe EB, Ijoma CK (2006). Left ventricular hypertrophy in African Black patients with chronic renal failure at first evaluation. Ethn Dis.

[13] Ojji D, Stewart S, Ajayi S, Manmak M, Sliwa K (2013). A predominance of hypertensive heart failure in the Abuja Heart Study cohort of urban Nigerians: a prospective clinical registry of 1515 de novo cases. Eur J Heart Fail.

[14] Sliwa K, Wilkinson D, Hansen C, Ntyintyane L, Tibazarwa K, Becker A (2008). Spectrum of heart disease and risk factors in a black urban population in South Africa (the Heart of Soweto Study): a cohort study. Lancet.

[15] Damasceno A, Mayosi BM, Sani M, Ogah OS, Mondo C, Ojji D (2012). The causes, treatment, and outcome of acute heart failure in 1006 Africans from 9 countries. Arch Intern Med.

[16] Kingue S, Dzudie A, Menanga A, Akono M, Ouankou M, Muna W (2005). A new look at adult chronic heart failure in Africa in the age of the Doppler echocardiography: experience of the medicine department at Yaounde General Hospital. Ann Cardiol Angeiol (Paris).

[17] Rodgers A, Ezzati M, Hoorn SV, Lopez AD, Lin RB, Murray CJ (2004). Distribution of major health risks: findings from the Global Burden of Disease Study. PLoS Med.

[18] Psaty BM, Lumley T, Furberg CD, Schellenbaum G, Pahor M, Alderman MH (2003). Health outcomes associated with various antihypertensive therapies used as first-line agents: a network meta-analysis. JAMA.

[19] Chobanian AV (2003). Joint National Committee on Prevention, Detection, Evaluation, and Treatment of High Blood Pressure. National Heart, Lung, and Blood Institute; National High Blood Pressure Education Program Coordinating Committee: seventh report of the Joint National Committee on Prevention, Detection, Evaluation, and Treatment of High Blood Pressure. Hypertension.

[20] Damasceno A, Dzudie A, Mayosi B (2007). Heart failure in sub-Saharan Africa: time of action. J Am Coll Cardiol.

[21] Habte B, Alemseqed F, Tesfaye D (2010). The pattern of cardiac diseases at the cardiac clinic of Jimma University specialised hospital, South West Ethiopia. Ethiop J Health Sci.

[22] Kengne AP, Amoah AG, Mbanya JC (2005). Cardiovascular complications of diabetes mellitus in sub-Saharan Africa. Circulation.

[23] Nkoke C, Luchuo EB (2016). Coronary heart disease in sub-Saharan Africa: still rare, misdiagnosed or underdiagnosed?. Cardiovasc Diagn Ther..

[24] Carapetis JR, McDonald M, Wilson NJ (2005). Acute rheumatic fever. Lancet.

[25] Carapetis JR, Steer AC, Mulholland EK, Weber M (2005). The global burden of group A streptococcal diseases. Lancet Infect Dis..

[26] Marijon E, Mirabel M, Celermajer DS, Jouven X (2012). Rheumatic heart disease. Lancet.

[27] Essop MR, Nkomo VT (2005). Rheumatic and nonrheumatic valvular heart disease: epidemiology, management, and prevention in Africa. Circulation.

